# Fevers

**Published:** 1905-09-02

**Authors:** 


### FEVERS.

(*Continued from page* 377.)

**Typhoid Fever.**—The impetus given to the study of typhoid fever in this country by the Boer War, and in the United States by the Spanish-American War of 1898, is not yet exhausted, as is abundantly evident when one considers how much space in current literature continues to be devoted to the subject, especially to the aspects of prevention and treatment. From Washington has been issued the Official Report[8] on the origin and spread of the fever in the United States military camps during the war with Spain. Its publication causes, to quote the words of a contemporary journal, "a feeling of almost sadness, since there is scarcely anything elicited which was not previously known by the profession," for "the dark history now revealed in all its ghastly details might have been prevented." The errors which have led to a preventable disease becoming the cause of such terrible suffering and waste of life seem bound up with the organisation of present military systems, and so far army administration has shown itself powerless to cope with them. One lesson, it is pointed out, must be learnt thoroughly in order to avoid a repetition of the "awful typhoid record of the Spanish War," and that is that disregard of the advice of the sanitary officers of the army should be treated as a grave military crime. Meanwhile, as another writer argues,[9] whatever the commonest channel of infection may be, it is of prime importance to endeavour to keep all channels free by preventing the dissemination of the bacillus typhosus. Efficient disinfection of the fæcal and urinary excretæ of all suffering from the disease will do this. According to Cole, strong carbolic acid is the surest agent where burning or boiling as of clothes, etc., is inadmissible. "Secondary cases," those apparently contracted one from another, have been numerous in a recent water-borne epidemic at Lincoln,[10] Dr. Klein's preliminary report of his investigations made for the Fishmongers' Company[11] shows that oysters readily take into their interior typhoid bacilli present in the surrounding water. They get rid of these again, probably by a process of devitalisation, more or less readily in proportion to the cleanliness of the water in which they have been, as well as are being, kept. Oysters which are "kept dry"—*i.e.* out of the water, especially those taken from polluted waters, get rid of the bacilli very much more slowly. In cockles, and still more readily in mussels, ingested bacteria show a decided increase in numbers which is followed by a very gradual and slow decrease.

**Treatment of Typhoid.**—Familiar as is the teaching of Dr. Ostler as to the necessity of giving water in unrestricted quantity to typhoid fever patients—and "more water" has been said to be a suitable motto for his typhoid wards—one cannot but be struck by the lengths to which this practice has been carried by Dr. E. F. Cushing[12] in the Lakeside Hospital, Ohio. By giving, during the waking hours, four ounces of water every 15 minutes in addition to the four-hourly alternate feeds of six ounces cf milk and lime water and four ounces af albumin water he has succeeded in securing the ingestion of from eight to fourteen pints of water in the 24 hours. This astonishing intake was accompanied by the daily elimination of from eight to twelve or more pints of watery urine. Contrary to what might have been expected the patients raised but little objection to the treatment, their general comfort was increased, and, in spite of the extra attentions required, the total nursing cares were lightened. Complications were diminished, and the symptoms generally, especially the toxic nervous symptoms, such as headache, apathy, restlessness, insomnia, and delirium were less marked, and baths less often required. Constipation was not, however, affected, and meteorism, that symptom of evil omen, was rather more common. The improvement appears to have been more marked in those cases in whom polyuria was greater—exceeding 160 oz. per diem. Of the 100 cases so treated the mortality was 5 per cent. as against one of 10·2 per cent. for the previous year, but it must be noted that most of the polyuria cases occurred during the decline of the epidemic, when its virulence was probably decreasing. The total daily excretion of chlorides was found to exceed the amount usually excreted by typhoid patients. In 1897 Dr. Owen Paget, of Freemantle, W. Australia, drew attention to the great value of olive oil given per rectum, and where possible by the mouth. Dr. E. H. Snell[13] has treated 100 consecutive cases in this way with one death. In one-fourth of these the Vidal reaction was sought for and found. From ½ to 1 oz. of the best olive oil was given every four hours on top of a little soda water and well borne. In addition, a daily enema of 8 to 10 oz. was usually given. The range of fever and the complications showed the cases were not of an exceptionally mild type. In another series of 116 cases Harbin[14] had a death-rate of 3·4 per cent. Therapeutic fasting of from one to three days and the withholding of milk were the main features of his treatment. Meat broths, egg albumin, and gelatine are in his view more readily digested, and therefore more suitable. Later his diet is more generous, as he recognises inanition may be a cause of prolonged temperature. That milk possesses many disadvantages for typhoid patients in spite of its great food value is generally admitted. Dr. Springthorpe,[15] of Melbourne, finds that a sterilised hopped malt extract is a very excellent substitute for milk or broths.

[8] Med. Rec., April 22. [9] Ibid., Jan. 7, p. 9. [10] Brit. Med. Jour., March 4, and April 15; Lancet, March 4. [11] Brit. Med. Jour., March 18, p. 611. [12] Amer. Jour. Med. Sc., Feb. [13] Brit. Med. Jour., Feb. 25, p. 414. [14] Scottish Med. Jour., April. [15] Lancet, April 8, p. 9[illegible]3.

